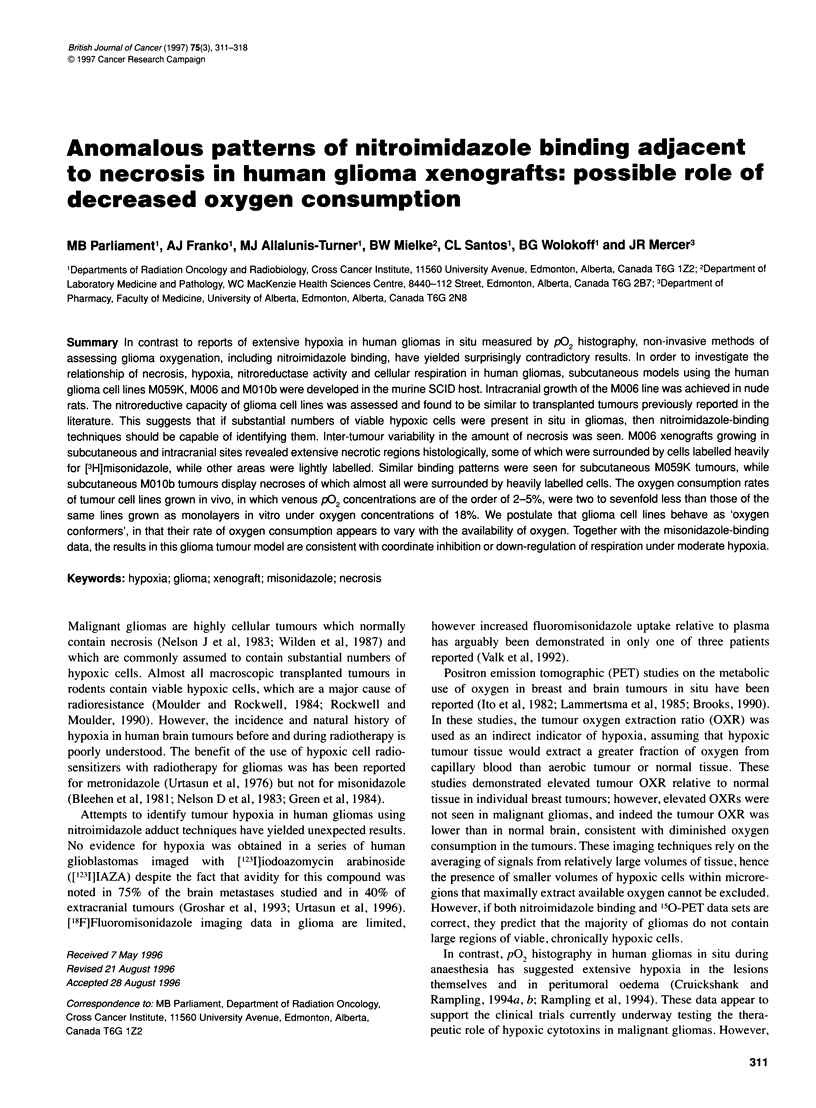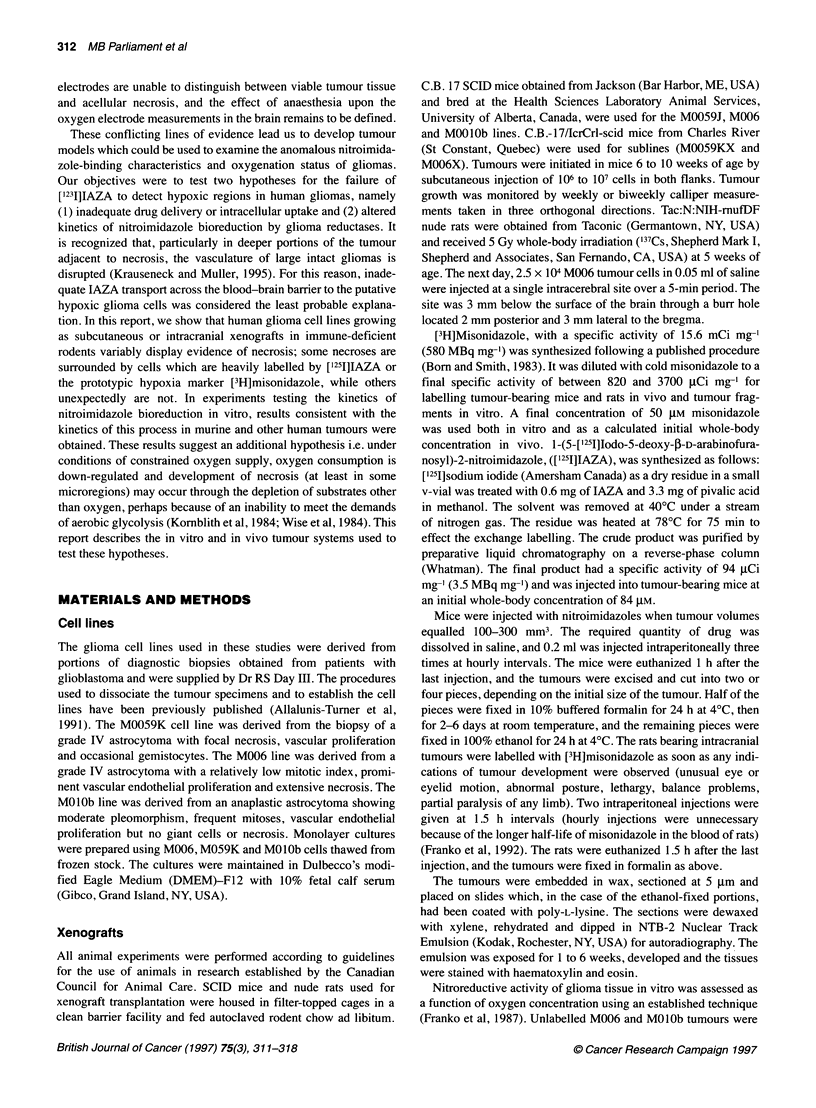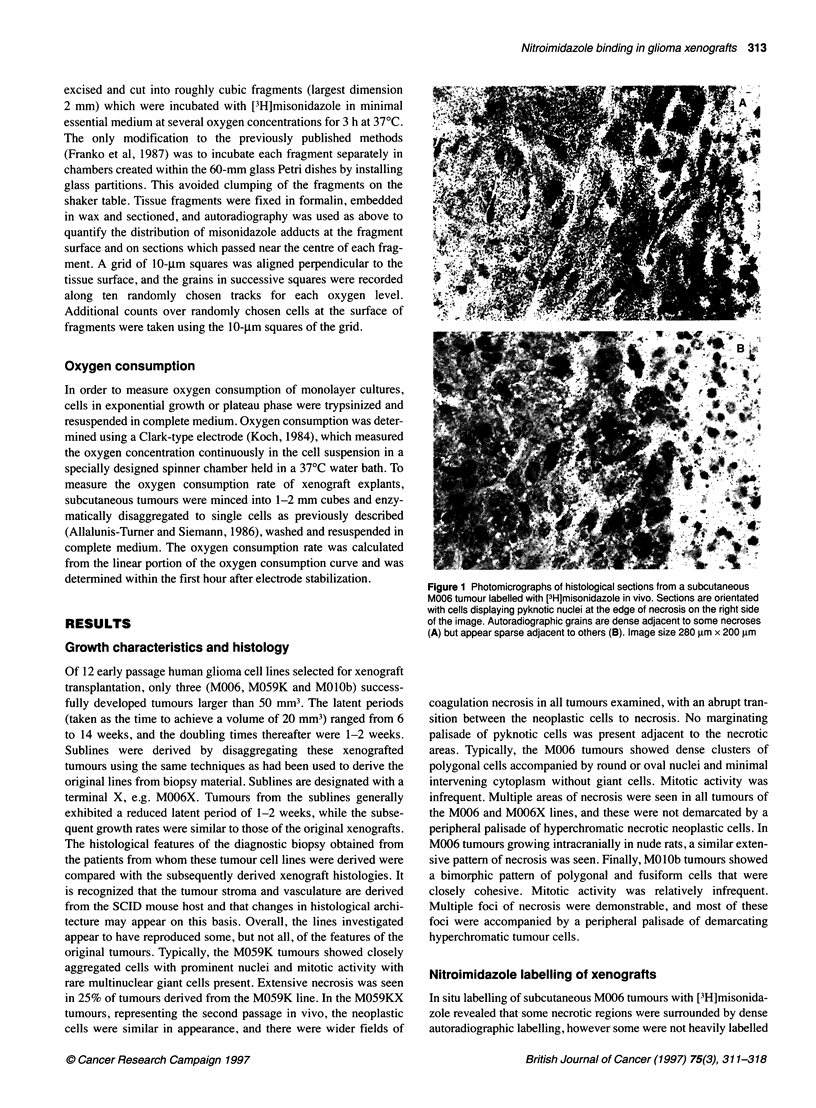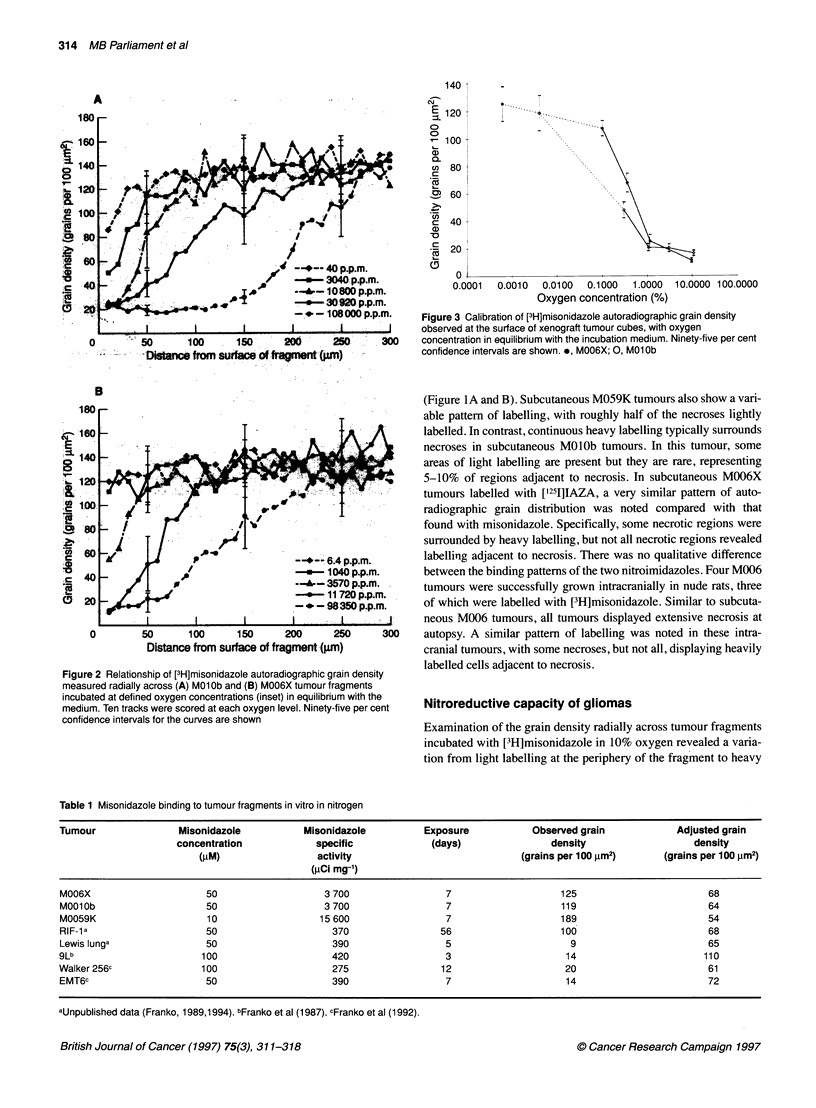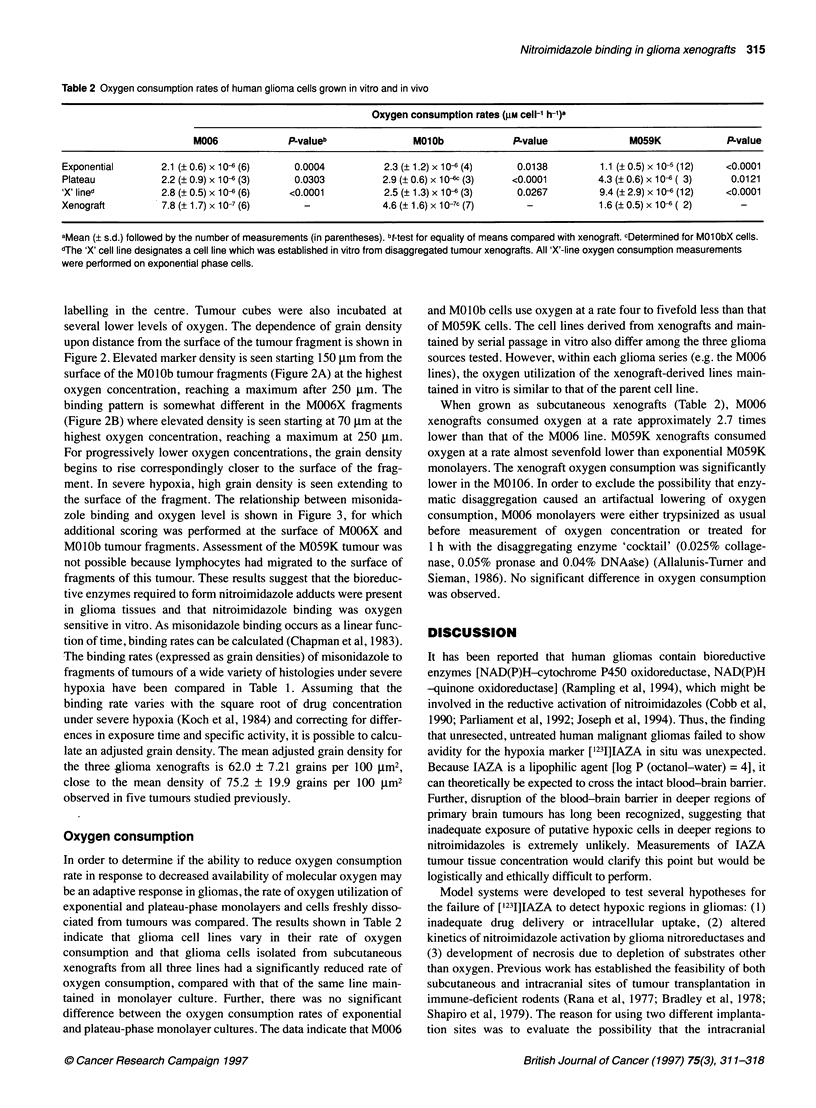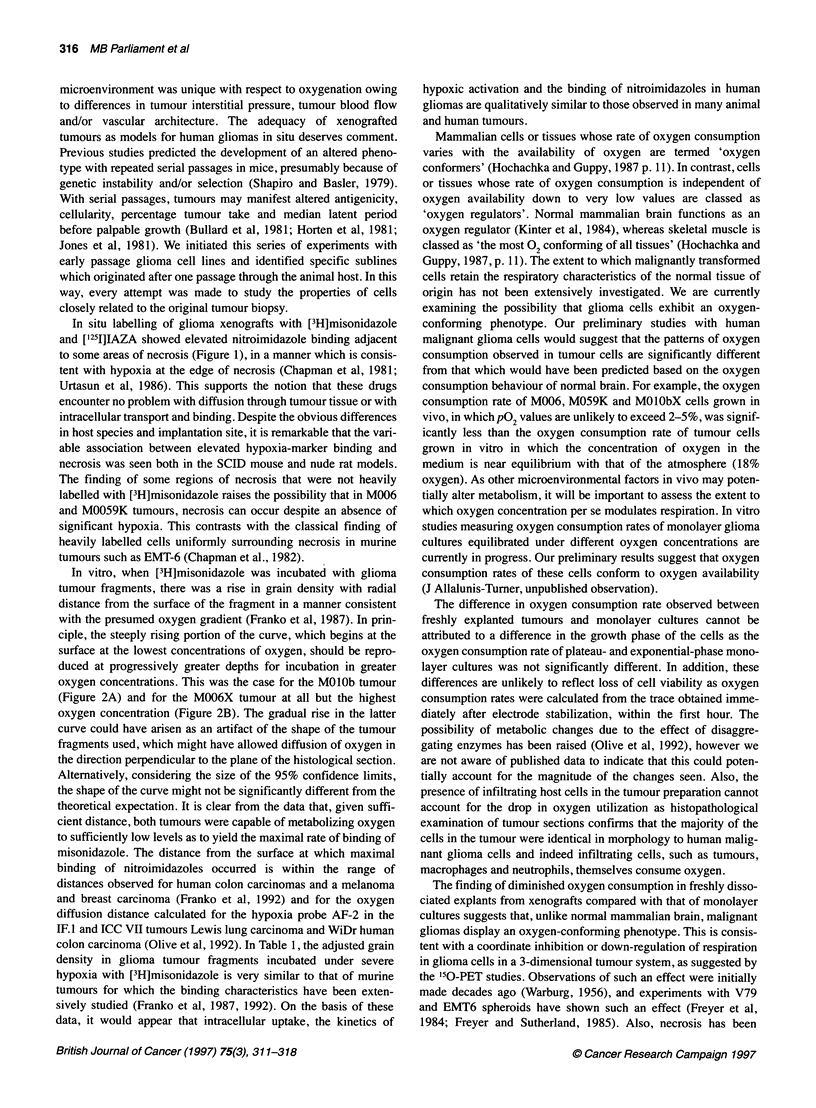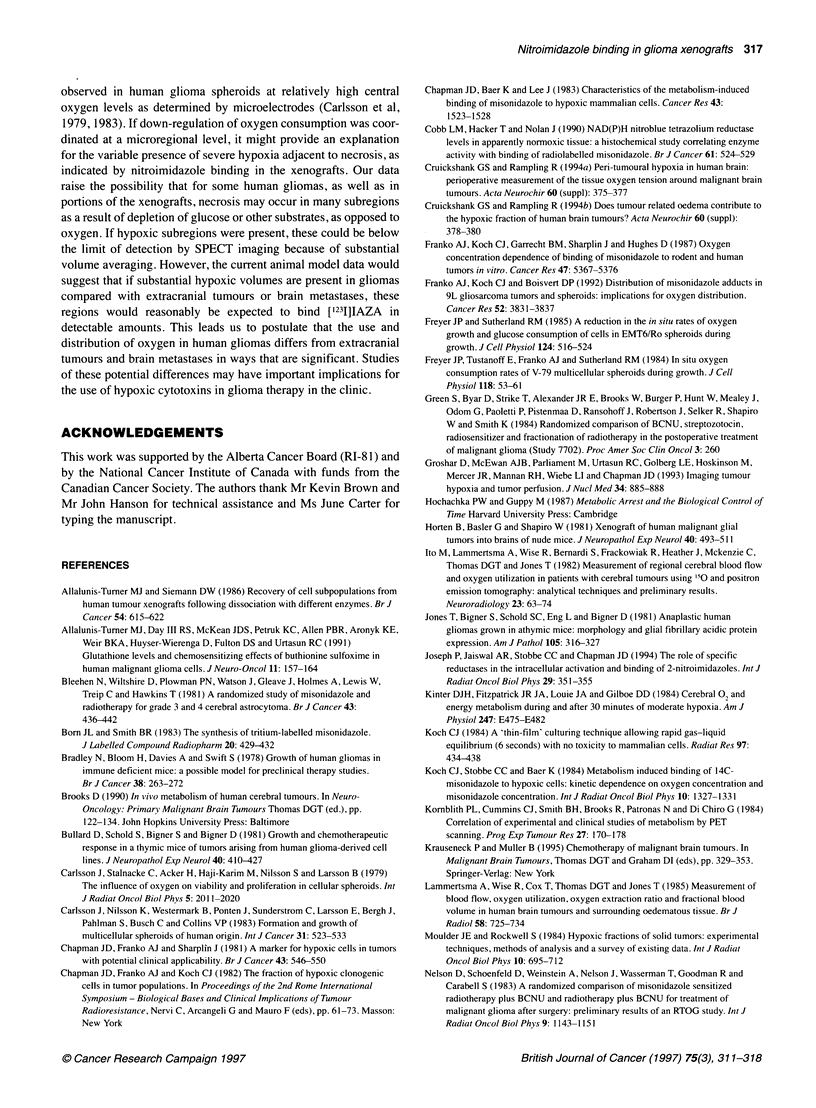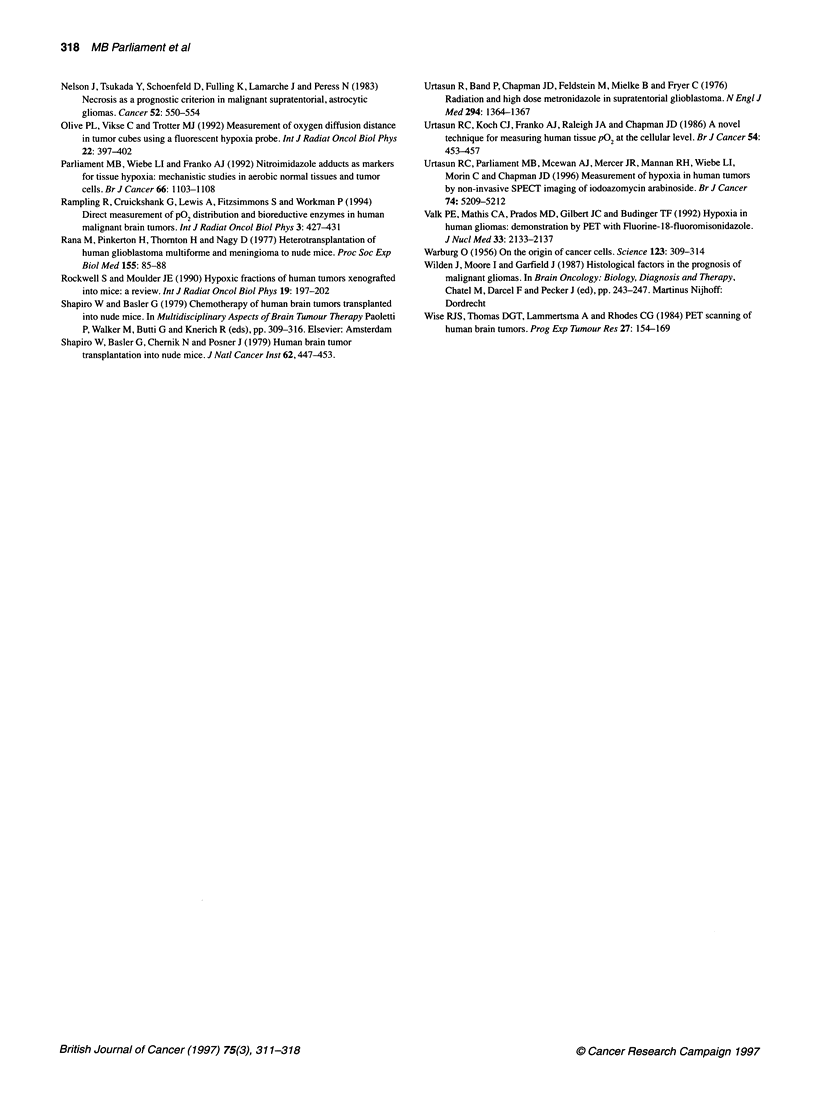# Anomalous patterns of nitroimidazole binding adjacent to necrosis in human glioma xenografts: possible role of decreased oxygen consumption.

**DOI:** 10.1038/bjc.1997.52

**Published:** 1997

**Authors:** M. B. Parliament, A. J. Franko, M. J. Allalunis-Turner, B. W. Mielke, C. L. Santos, B. G. Wolokoff, J. R. Mercer

**Affiliations:** Department of Radiation Oncology, Cross Cancer Institute, Edmonton, Alberta, Canada.

## Abstract

**Images:**


British Journal of Cancer (1997) 75(3), 311–318
© 1997 Cancer Research Campaign

### Anomalous patterns of nitroimidazole binding adjacent to necrosis in human glioma xenografts: possible role of decreased oxygen consumption

**MB Parliament[1], AJ Franko[1], MJ Allalunis-Turner[1], BW Mielke[2], CL Santos[1], BG Wolokoff[1] and JR Mercer[3]**

[1]Departments of Radiation Oncology and Radiobiology, Cross Cancer Institute, 11560 University Avenue, Edmonton, Alberta, Canada T6G 1Z2; [2]Department of Laboratory Medicine and Pathology, WC MacKenzie Health Sciences Centre, 8440–112 Street, Edmonton, Alberta, Canada T6G 2B7; [3]Department of Pharmacy, Faculty of Medicine, University of Alberta, Edmonton, Alberta, Canada T6G 2N8

**Summary** In contrast to reports of extensive hypoxia in human gliomas in situ measured by $pO_2$ histography, non-invasive methods of assessing glioma oxygenation, including nitroimidazole binding, have yielded surprisingly contradictory results. In order to investigate the relationship of necrosis, hypoxia, nitroreductase activity and cellular respiration in human gliomas, subcutaneous models using the human glioma cell lines M059K, M006 and M010b were developed in the murine SCID host. Intracranial growth of the M006 line was achieved in nude rats. The nitroreductive capacity of glioma cell lines was assessed and found to be similar to transplanted tumours previously reported in the literature. This suggests that if substantial numbers of viable hypoxic cells were present in situ in gliomas, then nitroimidazole-binding techniques should be capable of identifying them. Inter-tumour variability in the amount of necrosis was seen. M006 xenografts growing in subcutaneous and intracranial sites revealed extensive necrotic regions histologically, some of which were surrounded by cells labelled heavily for [$^3$H]misonidazole, while other areas were lightly labelled. Similar binding patterns were seen for subcutaneous M059K tumours, while subcutaneous M010b tumours display necroses of which almost all were surrounded by heavily labelled cells. The oxygen consumption rates of tumour cell lines grown in vivo, in which venous $pO_2$ concentrations are of the order of 2–5%, were two to sevenfold less than those of the same lines grown as monolayers in vitro under oxygen concentrations of 18%. We postulate that glioma cell lines behave as 'oxygen conformers', in that their rate of oxygen consumption appears to vary with the availability of oxygen. Together with the misonidazole-binding data, the results in this glioma tumour model are consistent with coordinate inhibition or down-regulation of respiration under moderate hypoxia.

**Keywords:** hypoxia; glioma; xenograft; misonidazole; necrosis

Malignant gliomas are highly cellular tumours which normally contain necrosis (Nelson J et al, 1983; Wilden et al, 1987) and which are commonly assumed to contain substantial numbers of hypoxic cells. Almost all macroscopic transplanted tumours in rodents contain viable hypoxic cells, which are a major cause of radioresistance (Moulder and Rockwell, 1984; Rockwell and Moulder, 1990). However, the incidence and natural history of hypoxia in human brain tumours before and during radiotherapy is poorly understood. The benefit of the use of hypoxic cell radiosensitizers with radiotherapy for gliomas was has been reported for metronidazole (Urtasun et al, 1976) but not for misonidazole (Bleehen et al, 1981; Nelson D et al, 1983; Green et al, 1984).

Attempts to identify tumour hypoxia in human gliomas using nitroimidazole adduct techniques have yielded unexpected results. No evidence for hypoxia was obtained in a series of human glioblastomas imaged with [$^{123}$I]iodoazomycin arabinoside ([$^{123}$I]IAZA) despite the fact that avidity for this compound was noted in 75% of the brain metastases studied and in 40% of extracranial tumours (Groshar et al, 1993; Urtasun et al, 1996). [$^{18}$F]Fluoromisonidazole imaging data in glioma are limited, however increased fluoromisonidazole uptake relative to plasma has arguably been demonstrated in only one of three patients reported (Valk et al, 1992).

Positron emission tomographic (PET) studies on the metabolic use of oxygen in breast and brain tumours in situ have been reported (Ito et al, 1982; Lammertsma et al, 1985; Brooks, 1990). In these studies, the tumour oxygen extraction ratio (OXR) was used as an indirect indicator of hypoxia, assuming that hypoxic tumour tissue would extract a greater fraction of oxygen from capillary blood than aerobic tumour or normal tissue. These studies demonstrated elevated tumour OXR relative to normal tissue in individual breast tumours; however, elevated OXRs were not seen in malignant gliomas, and indeed the tumour OXR was lower than in normal brain, consistent with diminished oxygen consumption in the tumours. These imaging techniques rely on the averaging of signals from relatively large volumes of tissue, hence the presence of smaller volumes of hypoxic cells within microregions that maximally extract available oxygen cannot be excluded. However, if both nitroimidazole binding and $^{15}$O-PET data sets are correct, they predict that the majority of gliomas do not contain large regions of viable, chronically hypoxic cells.

In contrast, $pO_2$ histography in human gliomas in situ during anaesthesia has suggested extensive hypoxia in the lesions themselves and in peritumoral oedema (Cruickshank and Rampling, 1994*a*, *b*; Rampling et al, 1994). These data appear to support the clinical trials currently underway testing the therapeutic role of hypoxic cytotoxins in malignant gliomas. However,

*Received 7 May 1996*
*Revised 21 August 1996*
*Accepted 28 August 1996*

*Correspondence to:* MB Parliament, Department of Radiation Oncology, Cross Cancer Institute, 11560 University Avenue, Edmonton, Alberta, Canada T6G 1Z2

electrodes are unable to distinguish between viable tumour tissue and acellular necrosis, and the effect of anaesthesia upon the oxygen electrode measurements in the brain remains to be defined.

These conflicting lines of evidence lead us to develop tumour models which could be used to examine the anomalous nitroimidazole-binding characteristics and oxygenation status of gliomas. Our objectives were to test two hypotheses for the failure of [$^{123}$I]IAZA to detect hypoxic regions in human gliomas, namely (1) inadequate drug delivery or intracellular uptake and (2) altered kinetics of nitroimidazole bioreduction by glioma reductases. It is recognized that, particularly in deeper portions of the tumour adjacent to necrosis, the vasculature of large intact gliomas is disrupted (Krauseneck and Muller, 1995). For this reason, inadequate IAZA transport across the blood–brain barrier to the putative hypoxic glioma cells was considered the least probable explanation. In this report, we show that human glioma cell lines growing as subcutaneous or intracranial xenografts in immune-deficient rodents variably display evidence of necrosis; some necroses are surrounded by cells which are heavily labelled by [$^{125}$I]IAZA or the prototypic hypoxia marker [$^{3}$H]misonidazole, while others unexpectedly are not. In experiments testing the kinetics of nitroimidazole bioreduction in vitro, results consistent with the kinetics of this process in murine and other human tumours were obtained. These results suggest an additional hypothesis i.e. under conditions of constrained oxygen supply, oxygen consumption is down-regulated and development of necrosis (at least in some microregions) may occur through the depletion of substrates other than oxygen, perhaps because of an inability to meet the demands of aerobic glycolysis (Kornblith et al, 1984; Wise et al, 1984). This report describes the in vitro and in vivo tumour systems used to test these hypotheses.

### MATERIALS AND METHODS

### Cell lines

The glioma cell lines used in these studies were derived from portions of diagnostic biopsies obtained from patients with glioblastoma and were supplied by Dr RS Day III. The procedures used to dissociate the tumour specimens and to establish the cell lines have been previously published (Allalunis-Turner et al, 1991). The M0059K cell line was derived from the biopsy of a grade IV astrocytoma with focal necrosis, vascular proliferation and occasional gemistocytes. The M006 line was derived from a grade IV astrocytoma with a relatively low mitotic index, prominent vascular endothelial proliferation and extensive necrosis. The M010b line was derived from an anaplastic astrocytoma showing moderate pleomorphism, frequent mitoses, vascular endothelial proliferation but no giant cells or necrosis. Monolayer cultures were prepared using M006, M059K and M010b cells thawed from frozen stock. The cultures were maintained in Dulbecco's modified Eagle Medium (DMEM)–F12 with 10% fetal calf serum (Gibco, Grand Island, NY, USA).

### Xenografts

All animal experiments were performed according to guidelines for the use of animals in research established by the Canadian Council for Animal Care. SCID mice and nude rats used for xenograft transplantation were housed in filter-topped cages in a clean barrier facility and fed autoclaved rodent chow ad libitum. C.B. 17 SCID mice obtained from Jackson (Bar Harbor, ME, USA) and bred at the Health Sciences Laboratory Animal Services, University of Alberta, Canada, were used for the M0059J, M006 and M0010b lines. C.B.-17/IcrCrl-scid mice from Charles River (St Constant, Quebec) were used for sublines (M0059KX and M006X). Tumours were initiated in mice 6 to 10 weeks of age by subcutaneous injection of $10^6$ to $10^7$ cells in both flanks. Tumour growth was monitored by weekly or biweekly calliper measurements taken in three orthogonal directions. Tac:N:NIH-rnufDF nude rats were obtained from Taconic (Germantown, NY, USA) and received 5 Gy whole-body irradiation ($^{137}$Cs, Shepherd Mark I, Shepherd and Associates, San Fernando, CA, USA) at 5 weeks of age. The next day, $2.5 \times 10^4$ M006 tumour cells in 0.05 ml of saline were injected at a single intracerebral site over a 5-min period. The site was 3 mm below the surface of the brain through a burr hole located 2 mm posterior and 3 mm lateral to the bregma.

[$^{3}$H]Misonidazole, with a specific activity of 15.6 mCi mg$^{-1}$ (580 MBq mg$^{-1}$) was synthesized following a published procedure (Born and Smith, 1983). It was diluted with cold misonidazole to a final specific activity of between 820 and 3700 μCi mg$^{-1}$ for labelling tumour-bearing mice and rats in vivo and tumour fragments in vitro. A final concentration of 50 μM misonidazole was used both in vitro and as a calculated initial whole-body concentration in vivo. 1-(5-[$^{125}$I]Iodo-5-deoxy-β-D-arabinofuranosyl)-2-nitroimidazole, ([$^{125}$I]IAZA), was synthesized as follows: [$^{125}$I]sodium iodide (Amersham Canada) as a dry residue in a small v-vial was treated with 0.6 mg of IAZA and 3.3 mg of pivalic acid in methanol. The solvent was removed at 40°C under a stream of nitrogen gas. The residue was heated at 78°C for 75 min to effect the exchange labelling. The crude product was purified by preparative liquid chromatography on a reverse-phase column (Whatman). The final product had a specific activity of 94 μCi mg$^{-1}$ (3.5 MBq mg$^{-1}$) and was injected into tumour-bearing mice at an initial whole-body concentration of 84 μM.

Mice were injected with nitroimidazoles when tumour volumes equalled 100–300 mm$^3$. The required quantity of drug was dissolved in saline, and 0.2 ml was injected intraperitoneally three times at hourly intervals. The mice were euthanized 1 h after the last injection, and the tumours were excised and cut into two or four pieces, depending on the initial size of the tumour. Half of the pieces were fixed in 10% buffered formalin for 24 h at 4°C, then for 2–6 days at room temperature, and the remaining pieces were fixed in 100% ethanol for 24 h at 4°C. The rats bearing intracranial tumours were labelled with [$^{3}$H]misonidazole as soon as any indications of tumour development were observed (unusual eye or eyelid motion, abnormal posture, lethargy, balance problems, partial paralysis of any limb). Two intraperitoneal injections were given at 1.5 h intervals (hourly injections were unnecessary because of the longer half-life of misonidazole in the blood of rats) (Franko et al, 1992). The rats were euthanized 1.5 h after the last injection, and the tumours were fixed in formalin as above.

The tumours were embedded in wax, sectioned at 5 μm and placed on slides which, in the case of the ethanol-fixed portions, had been coated with poly-L-lysine. The sections were dewaxed with xylene, rehydrated and dipped in NTB-2 Nuclear Track Emulsion (Kodak, Rochester, NY, USA) for autoradiography. The emulsion was exposed for 1 to 6 weeks, developed and the tissues were stained with haematoxylin and eosin.

Nitroreductive activity of glioma tissue in vitro was assessed as a function of oxygen concentration using an established technique (Franko et al, 1987). Unlabelled M006 and M010b tumours were

© Cancer Research Campaign 1997

excised and cut into roughly cubic fragments (largest dimension 2 mm) which were incubated with [$^3$H]misonidazole in minimal essential medium at several oxygen concentrations for 3 h at 37°C. The only modification to the previously published methods (Franko et al, 1987) was to incubate each fragment separately in chambers created within the 60-mm glass Petri dishes by installing glass partitions. This avoided clumping of the fragments on the shaker table. Tissue fragments were fixed in formalin, embedded in wax and sectioned, and autoradiography was used as above to quantify the distribution of misonidazole adducts at the fragment surface and on sections which passed near the centre of each fragment. A grid of 10-μm squares was aligned perpendicular to the tissue surface, and the grains in successive squares were recorded along ten randomly chosen tracks for each oxygen level. Additional counts over randomly chosen cells at the surface of fragments were taken using the 10-μm squares of the grid.

### Oxygen consumption

In order to measure oxygen consumption of monolayer cultures, cells in exponential growth or plateau phase were trypsinized and resuspended in complete medium. Oxygen consumption was determined using a Clark-type electrode (Koch, 1984), which measured the oxygen concentration continuously in the cell suspension in a specially designed spinner chamber held in a 37°C water bath. To measure the oxygen consumption rate of xenograft explants, subcutaneous tumours were minced into 1–2 mm cubes and enzymatically disaggregated to single cells as previously described (Allalunis-Turner and Siemann, 1986), washed and resuspended in complete medium. The oxygen consumption rate was calculated from the linear portion of the oxygen consumption curve and was determined within the first hour after electrode stabilization.

### RESULTS

### Growth characteristics and histology

Of 12 early passage human glioma cell lines selected for xenograft transplantation, only three (M006, M059K and M010b) successfully developed tumours larger than 50 mm$^3$. The latent periods (taken as the time to achieve a volume of 20 mm$^3$) ranged from 6 to 14 weeks, and the doubling times thereafter were 1–2 weeks. Sublines were derived by disaggregating these xenografted tumours using the same techniques as had been used to derive the original lines from biopsy material. Sublines are designated with a terminal X, e.g. M006X. Tumours from the sublines generally exhibited a reduced latent period of 1–2 weeks, while the subsequent growth rates were similar to those of the original xenografts. The histological features of the diagnostic biopsy obtained from the patients from whom these tumour cell lines were derived were compared with the subsequently derived xenograft histologies. It is recognized that the tumour stroma and vasculature are derived from the SCID mouse host and that changes in histological architecture may appear on this basis. Overall, the lines investigated appear to have reproduced some, but not all, of the features of the original tumours. Typically, the M059K tumours showed closely aggregated cells with prominent nuclei and mitotic activity with rare multinuclear giant cells present. Extensive necrosis was seen in 25% of tumours derived from the M059K line. In the M059KX tumours, representing the second passage in vivo, the neoplastic cells were similar in appearance, and there were wider fields of coagulation necrosis in all tumours examined, with an abrupt transition between the neoplastic cells to necrosis. No marginating palisade of pyknotic cells was present adjacent to the necrotic areas. Typically, the M006 tumours showed dense clusters of polygonal cells accompanied by round or oval nuclei and minimal intervening cytoplasm without giant cells. Mitotic activity was infrequent. Multiple areas of necrosis were seen in all tumours of the M006 and M006X lines, and these were not demarcated by a peripheral palisade of hyperchromatic necrotic neoplastic cells. In M006 tumours growing intracranially in nude rats, a similar extensive pattern of necrosis was seen. Finally, M010b tumours showed a bimorphic pattern of polygonal and fusiform cells that were closely cohesive. Mitotic activity was relatively infrequent. Multiple foci of necrosis were demonstrable, and most of these foci were accompanied by a peripheral palisade of demarcating hyperchromatic tumour cells.

**Figure 1** Photomicrographs of histological sections from a subcutaneous M006 tumour labelled with [$^3$H]misonidazole in vivo. Sections are orientated with cells displaying pyknotic nuclei at the edge of necrosis on the right side of the image. Autoradiographic grains are dense adjacent to some necroses (**A**) but appear sparse adjacent to others (**B**). Image size 280 μm × 200 μm

### Nitroimidazole labelling of xenografts

In situ labelling of subcutaneous M006 tumours with [$^3$H]misonidazole revealed that some necrotic regions were surrounded by dense autoradiographic labelling, however some were not heavily labelled

© Cancer Research Campaign 1997

**Figure 2** Relationship of [$^{3}$H]misonidazole autoradiographic grain density measured radially across (**A**) M010b and (**B**) M006X tumour fragments incubated at defined oxygen concentrations (inset) in equilibrium with the medium. Ten tracks were scored at each oxygen level. Ninety-five per cent confidence intervals for the curves are shown

**Figure 3** Calibration of [$^{3}$H]misonidazole autoradiographic grain density observed at the surface of xenograft tumour cubes, with oxygen concentration in equilibrium with the incubation medium. Ninety-five per cent confidence intervals are shown. ●, M006X; O, M010b

(Figure 1A and B). Subcutaneous M059K tumours also show a variable pattern of labelling, with roughly half of the necroses lightly labelled. In contrast, continuous heavy labelling typically surrounds necroses in subcutaneous M010b tumours. In this tumour, some areas of light labelling are present but they are rare, representing 5–10% of regions adjacent to necrosis. In subcutaneous M006X tumours labelled with [$^{125}$I]IAZA, a very similar pattern of autoradiographic grain distribution was noted compared with that found with misonidazole. Specifically, some necrotic regions were surrounded by heavy labelling, but not all necrotic regions revealed labelling adjacent to necrosis. There was no qualitative difference between the binding patterns of the two nitroimidazoles. Four M006 tumours were successfully grown intracranially in nude rats, three of which were labelled with [$^{3}$H]misonidazole. Similar to subcutaneous M006 tumours, all tumours displayed extensive necrosis at autopsy. A similar pattern of labelling was noted in these intracranial tumours, with some necroses, but not all, displaying heavily labelled cells adjacent to necrosis.

### Nitroreductive capacity of gliomas

Examination of the grain density radially across tumour fragments incubated with [$^{3}$H]misonidazole in 10% oxygen revealed a variation from light labelling at the periphery of the fragment to heavy

**Table 1** Misonidazole binding to tumour fragments in vitro in nitrogen

| Tumour | Misonidazole concentration (µM) | Misonidazole specific activity (µCi mg$^{-1}$) | Exposure (days) | Observed grain density (grains per 100 µm$^{2}$) | Adjusted grain density (grains per 100 µm$^{2}$) |
|---|---|---|---|---|---|
| M006X | 50 | 3 700 | 7 | 125 | 68 |
| M0010b | 50 | 3 700 | 7 | 119 | 64 |
| M0059K | 10 | 15 600 | 7 | 189 | 54 |
| RIF-1[a] | 50 | 370 | 56 | 100 | 68 |
| Lewis lung[a] | 50 | 390 | 5 | 9 | 65 |
| 9L[b] | 100 | 420 | 3 | 14 | 110 |
| Walker 256[c] | 100 | 275 | 12 | 20 | 61 |
| EMT6[c] | 50 | 390 | 7 | 14 | 72 |

[a]Unpublished data (Franko, 1989,1994). [b]Franko et al (1987). [c]Franko et al (1992).

 © Cancer Research Campaign 1997

**Table 2** Oxygen consumption rates of human glioma cells grown in vitro and in vivo

| | Oxygen consumption rates (μM $cell^{-1}$ $h^{-1}$)[a] | | | | | |
|---|---|---|---|---|---|---|
| | **M006** | ***P*-value**[b] | **M010b** | ***P*-value** | **M059K** | ***P*-value** |
| Exponential | 2.1 (± 0.6) × $10^{-6}$ (6) | 0.0004 | 2.3 (± 1.2) × $10^{-6}$ (4) | 0.0138 | 1.1 (± 0.5) × $10^{-5}$ (12) | <0.0001 |
| Plateau | 2.2 (± 0.9) × $10^{-6}$ (3) | 0.0303 | 2.9 (± 0.6) × $10^{-6}$[c] (3) | <0.0001 | 4.3 (± 0.6) × $10^{-6}$ ( 3) | 0.0121 |
| 'X' line[d] | 2.8 (± 0.5) × $10^{-6}$ (6) | <0.0001 | 2.5 (± 1.3) × $10^{-6}$ (3) | 0.0267 | 9.4 (± 2.9) × $10^{-6}$ (12) | <0.0001 |
| Xenograft | 7.8 (± 1.7) × $10^{-7}$ (6) | – | 4.6 (± 1.6) × $10^{-7}$[c] (7) | – | 1.6 (± 0.5) × $10^{-6}$ ( 2) | – |

[a]Mean (± s.d.) followed by the number of measurements (in parentheses). [b]*t*-test for equality of means compared with xenograft. [c]Determined for M010bX cells. [d]The 'X' cell line designates a cell line which was established in vitro from disaggregated tumour xenografts. All 'X'-line oxygen consumption measurements were performed on exponential phase cells.

labelling in the centre. Tumour cubes were also incubated at several lower levels of oxygen. The dependence of grain density upon distance from the surface of the tumour fragment is shown in Figure 2. Elevated marker density is seen starting 150 μm from the surface of the M010b tumour fragments (Figure 2A) at the highest oxygen concentration, reaching a maximum after 250 μm. The binding pattern is somewhat different in the M006X fragments (Figure 2B) where elevated density is seen starting at 70 μm at the highest oxygen concentration, reaching a maximum at 250 μm. For progressively lower oxygen concentrations, the grain density begins to rise correspondingly closer to the surface of the fragment. In severe hypoxia, high grain density is seen extending to the surface of the fragment. The relationship between misonidazole binding and oxygen level is shown in Figure 3, for which additional scoring was performed at the surface of M006X and M010b tumour fragments. Assessment of the M059K tumour was not possible because lymphocytes had migrated to the surface of fragments of this tumour. These results suggest that the bioreductive enzymes required to form nitroimidazole adducts were present in glioma tissues and that nitroimidazole binding was oxygen sensitive in vitro. As misonidazole binding occurs as a linear function of time, binding rates can be calculated (Chapman et al, 1983). The binding rates (expressed as grain densities) of misonidazole to fragments of tumours of a wide variety of histologies under severe hypoxia have been compared in Table 1. Assuming that the binding rate varies with the square root of drug concentration under severe hypoxia (Koch et al, 1984) and correcting for differences in exposure time and specific activity, it is possible to calculate an adjusted grain density. The mean adjusted grain density for the three glioma xenografts is 62.0 ± 7.21 grains per 100 $\mu m^2$, close to the mean density of 75.2 ± 19.9 grains per 100 $\mu m^2$ observed in five tumours studied previously.

### Oxygen consumption

In order to determine if the ability to reduce oxygen consumption rate in response to decreased availability of molecular oxygen may be an adaptive response in gliomas, the rate of oxygen utilization of exponential and plateau-phase monolayers and cells freshly dissociated from tumours was compared. The results shown in Table 2 indicate that glioma cell lines vary in their rate of oxygen consumption and that glioma cells isolated from subcutaneous xenografts from all three lines had a significantly reduced rate of oxygen consumption, compared with that of the same line maintained in monolayer culture. Further, there was no significant difference between the oxygen consumption rates of exponential and plateau-phase monolayer cultures. The data indicate that M006 and M010b cells use oxygen at a rate four to fivefold less than that of M059K cells. The cell lines derived from xenografts and maintained by serial passage in vitro also differ among the three glioma sources tested. However, within each glioma series (e.g. the M006 lines), the oxygen utilization of the xenograft-derived lines maintained in vitro is similar to that of the parent cell line.

When grown as subcutaneous xenografts (Table 2), M006 xenografts consumed oxygen at a rate approximately 2.7 times lower than that of the M006 line. M059K xenografts consumed oxygen at a rate almost sevenfold lower than exponential M059K monolayers. The xenograft oxygen consumption was significantly lower in the M0106. In order to exclude the possibility that enzymatic disaggregation caused an artifactual lowering of oxygen consumption, M006 monolayers were either trypsinized as usual before measurement of oxygen concentration or treated for 1 h with the disaggregating enzyme 'cocktail' (0.025% collagenase, 0.05% pronase and 0.04% DNAa'se) (Allalunis-Turner and Sieman, 1986). No significant difference in oxygen consumption was observed.

### DISCUSSION

It has been reported that human gliomas contain bioreductive enzymes [NAD(P)H–cytochrome P450 oxidoreductase, NAD(P)H –quinone oxidoreductase] (Rampling et al, 1994), which might be involved in the reductive activation of nitroimidazoles (Cobb et al, 1990; Parliament et al, 1992; Joseph et al, 1994). Thus, the finding that unresected, untreated human malignant gliomas failed to show avidity for the hypoxia marker [$^{123}$I]IAZA in situ was unexpected. Because IAZA is a lipophilic agent [log P (octanol–water) = 4], it can theoretically be expected to cross the intact blood–brain barrier. Further, disruption of the blood–brain barrier in deeper regions of primary brain tumours has long been recognized, suggesting that inadequate exposure of putative hypoxic cells in deeper regions to nitroimidazoles is extremely unlikely. Measurements of IAZA tumour tissue concentration would clarify this point but would be logistically and ethically difficult to perform.

Model systems were developed to test several hypotheses for the failure of [$^{123}$I]IAZA to detect hypoxic regions in gliomas: (1) inadequate drug delivery or intracellular uptake, (2) altered kinetics of nitroimidazole activation by glioma nitroreductases and (3) development of necrosis due to depletion of substrates other than oxygen. Previous work has established the feasibility of both subcutaneous and intracranial sites of tumour transplantation in immune-deficient rodents (Rana et al, 1977; Bradley et al, 1978; Shapiro et al, 1979). The reason for using two different implantation sites was to evaluate the possibility that the intracranial

© Cancer Research Campaign 1997 

microenvironment was unique with respect to oxygenation owing to differences in tumour interstitial pressure, tumour blood flow and/or vascular architecture. The adequacy of xenografted tumours as models for human gliomas in situ deserves comment. Previous studies predicted the development of an altered phenotype with repeated serial passages in mice, presumably because of genetic instability and/or selection (Shapiro and Basler, 1979). With serial passages, tumours may manifest altered antigenicity, cellularity, percentage tumour take and median latent period before palpable growth (Bullard et al, 1981; Horten et al, 1981; Jones et al, 1981). We initiated this series of experiments with early passage glioma cell lines and identified specific sublines which originated after one passage through the animal host. In this way, every attempt was made to study the properties of cells closely related to the original tumour biopsy.

In situ labelling of glioma xenografts with [$^{3}$H]misonidazole and [$^{125}$I]IAZA showed elevated nitroimidazole binding adjacent to some areas of necrosis (Figure 1), in a manner which is consistent with hypoxia at the edge of necrosis (Chapman et al, 1981; Urtasun et al, 1986). This supports the notion that these drugs encounter no problem with diffusion through tumour tissue or with intracellular transport and binding. Despite the obvious differences in host species and implantation site, it is remarkable that the variable association between elevated hypoxia-marker binding and necrosis was seen both in the SCID mouse and nude rat models. The finding of some regions of necrosis that were not heavily labelled with [$^{3}$H]misonidazole raises the possibility that in M006 and M0059K tumours, necrosis can occur despite an absence of significant hypoxia. This contrasts with the classical finding of heavily labelled cells uniformly surrounding necrosis in murine tumours such as EMT-6 (Chapman et al., 1982).

In vitro, when [$^{3}$H]misonidazole was incubated with glioma tumour fragments, there was a rise in grain density with radial distance from the surface of the fragment in a manner consistent with the presumed oxygen gradient (Franko et al, 1987). In principle, the steeply rising portion of the curve, which begins at the surface at the lowest concentrations of oxygen, should be reproduced at progressively greater depths for incubation in greater oxygen concentrations. This was the case for the M010b tumour (Figure 2A) and for the M006X tumour at all but the highest oxygen concentration (Figure 2B). The gradual rise in the latter curve could have arisen as an artifact of the shape of the tumour fragments used, which might have allowed diffusion of oxygen in the direction perpendicular to the plane of the histological section. Alternatively, considering the size of the 95% confidence limits, the shape of the curve might not be significantly different from the theoretical expectation. It is clear from the data that, given sufficient distance, both tumours were capable of metabolizing oxygen to sufficiently low levels as to yield the maximal rate of binding of misonidazole. The distance from the surface at which maximal binding of nitroimidazoles occurred is within the range of distances observed for human colon carcinomas and a melanoma and breast carcinoma (Franko et al, 1992) and for the oxygen diffusion distance calculated for the hypoxia probe AF-2 in the IF.1 and ICC VII tumours Lewis lung carcinoma and WiDr human colon carcinoma (Olive et al, 1992). In Table 1, the adjusted grain density in glioma tumour fragments incubated under severe hypoxia with [$^{3}$H]misonidazole is very similar to that of murine tumours for which the binding characteristics have been extensively studied (Franko et al, 1987, 1992). On the basis of these data, it would appear that intracellular uptake, the kinetics of hypoxic activation and the binding of nitroimidazoles in human gliomas are qualitatively similar to those observed in many animal and human tumours.

Mammalian cells or tissues whose rate of oxygen consumption varies with the availability of oxygen are termed 'oxygen conformers' (Hochachka and Guppy, 1987 p. 11). In contrast, cells or tissues whose rate of oxygen consumption is independent of oxygen availability down to very low values are classed as 'oxygen regulators'. Normal mammalian brain functions as an oxygen regulator (Kinter et al, 1984), whereas skeletal muscle is classed as 'the most $O_2$ conforming of all tissues' (Hochachka and Guppy, 1987, p. 11). The extent to which malignantly transformed cells retain the respiratory characteristics of the normal tissue of origin has not been extensively investigated. We are currently examining the possibility that glioma cells exhibit an oxygen-conforming phenotype. Our preliminary studies with human malignant glioma cells would suggest that the patterns of oxygen consumption observed in tumour cells are significantly different from that which would have been predicted based on the oxygen consumption behaviour of normal brain. For example, the oxygen consumption rate of M006, M059K and M010bX cells grown in vivo, in which $pO_2$ values are unlikely to exceed 2–5%, was significantly less than the oxygen consumption rate of tumour cells grown in vitro in which the concentration of oxygen in the medium is near equilibrium with that of the atmosphere (18% oxygen). As other microenvironmental factors in vivo may potentially alter metabolism, it will be important to assess the extent to which oxygen concentration per se modulates respiration. In vitro studies measuring oxygen consumption rates of monolayer glioma cultures equilibrated under different oyxgen concentrations are currently in progress. Our preliminary results suggest that oxygen consumption rates of these cells conform to oxygen availability (J Allalunis-Turner, unpublished observation).

The difference in oxygen consumption rate observed between freshly explanted tumours and monolayer cultures cannot be attributed to a difference in the growth phase of the cells as the oxygen consumption rate of plateau- and exponential-phase monolayer cultures was not significantly different. In addition, these differences are unlikely to reflect loss of cell viability as oxygen consumption rates were calculated from the trace obtained immediately after electrode stabilization, within the first hour. The possibility of metabolic changes due to the effect of disaggregating enzymes has been raised (Olive et al, 1992), however we are not aware of published data to indicate that this could potentially account for the magnitude of the changes seen. Also, the presence of infiltrating host cells in the tumour preparation cannot account for the drop in oxygen utilization as histopathological examination of tumour sections confirms that the majority of the cells in the tumour were identical in morphology to human malignant glioma cells and indeed infiltrating cells, such as tumours, macrophages and neutrophils, themselves consume oxygen.

The finding of diminished oxygen consumption in freshly dissociated explants from xenografts compared with that of monolayer cultures suggests that, unlike normal mammalian brain, malignant gliomas display an oxygen-conforming phenotype. This is consistent with a coordinate inhibition or down-regulation of respiration in glioma cells in a 3-dimensional tumour system, as suggested by the $^{15}$O-PET studies. Observations of such an effect were initially made decades ago (Warburg, 1956), and experiments with V79 and EMT6 spheroids have shown such an effect (Freyer et al, 1984; Freyer and Sutherland, 1985). Also, necrosis has been

 © Cancer Research Campaign 1997

observed in human glioma spheroids at relatively high central oxygen levels as determined by microelectrodes (Carlsson et al, 1979, 1983). If down-regulation of oxygen consumption was coordinated at a microregional level, it might provide an explanation for the variable presence of severe hypoxia adjacent to necrosis, as indicated by nitroimidazole binding in the xenografts. Our data raise the possibility that for some human gliomas, as well as in portions of the xenografts, necrosis may occur in many subregions as a result of depletion of glucose or other substrates, as opposed to oxygen. If hypoxic subregions were present, these could be below the limit of detection by SPECT imaging because of substantial volume averaging. However, the current animal model data would suggest that if substantial hypoxic volumes are present in gliomas compared with extracranial tumours or brain metastases, these regions would reasonably be expected to bind [$^{123}$I]IAZA in detectable amounts. This leads us to postulate that the use and distribution of oxygen in human gliomas differs from extracranial tumours and brain metastases in ways that are significant. Studies of these potential differences may have important implications for the use of hypoxic cytotoxins in glioma therapy in the clinic.

### ACKNOWLEDGEMENTS

This work was supported by the Alberta Cancer Board (RI-81) and by the National Cancer Institute of Canada with funds from the Canadian Cancer Society. The authors thank Mr Kevin Brown and Mr John Hanson for technical assistance and Ms June Carter for typing the manuscript.

### REFERENCES

Allalunis-Turner MJ and Siemann DW (1986) Recovery of cell subpopulations from human tumour xenografts following dissociation with different enzymes. *Br J Cancer* **54**: 615–622

Allalunis-Turner MJ, Day III RS, McKean JDS, Petruk KC, Allen PBR, Aronyk KE, Weir BKA, Huyser-Wierenga D, Fulton DS and Urtasun RC (1991) Glutathione levels and chemosensitizing effects of buthionine sulfoxime in human malignant glioma cells. *J Neuro-Oncol* **11**: 157–164

Bleehen N, Wiltshire D, Plowman PN, Watson J, Gleave J, Holmes A, Lewis W, Treip C and Hawkins T (1981) A randomized study of misonidazole and radiotherapy for grade 3 and 4 cerebral astrocytoma. *Br J Cancer* **43**: 436–442

Born JL and Smith BR (1983) The synthesis of tritium-labelled misonidazole. *J Labelled Compound Radiopharm* **20**: 429–432

Bradley N, Bloom H, Davies A and Swift S (1978) Growth of human gliomas in immune deficient mice: a possible model for preclinical therapy studies. *Br J Cancer* **38**: 263–272

Brooks D (1990) *In vivo* metabolism of human cerebral tumours. In *Neuro-Oncology: Primary Malignant Brain Tumours* Thomas DGT (ed.), pp. 122–134. John Hopkins University Press: Baltimore

Bullard D, Schold S, Bigner S and Bigner D (1981) Growth and chemotherapeutic response in a thymic mice of tumors arising from human glioma-derived cell lines. *J Neuropathol Exp Neurol* **40**: 410–427

Carlsson J, Stalnacke C, Acker H, Haji-Karim M, Nilsson S and Larsson B (1979) The influence of oxygen on viability and proliferation in cellular spheroids. *Int J Radiat Oncol Biol Phys* **5**: 2011–2020

Carlsson J, Nilsson K, Westermark B, Ponten J, Sunderstrom C, Larsson E, Bergh J, Pahlman S, Busch C and Collins VP (1983) Formation and growth of multicellular spheroids of human origin. *Int J Cancer* **31**: 523–533

Chapman JD, Franko AJ and Sharplin J (1981) A marker for hypoxic cells in tumors with potential clinical applicability. *Br J Cancer* **43**: 546–550

Chapman JD, Franko AJ and Koch CJ (1982) The fraction of hypoxic clonogenic cells in tumor populations. In *Proceedings of the 2nd Rome International Symposium – Biological Bases and Clinical Implications of Tumour Radioresistance*, Nervi C, Arcangeli G and Mauro F (eds), pp. 61–73. Masson: New York

Chapman JD, Baer K and Lee J (1983) Characteristics of the metabolism-induced binding of misonidazole to hypoxic mammalian cells. *Cancer Res* **43**: 1523–1528

Cobb LM, Hacker T and Nolan J (1990) NAD(P)H nitroblue tetrazolium reductase levels in apparently normoxic tissue: a histochemical study correlating enzyme activity with binding of radiolabelled misonidazole. *Br J Cancer* **61**: 524–529

Cruickshank GS and Rampling R (1994*a*) Peri-tumoural hypoxia in human brain: perioperative measurement of the tissue oxygen tension around malignant brain tumours. *Acta Neurochir* **60** (suppl): 375–377

Cruickshank GS and Rampling R (1994*b*) Does tumour related oedema contribute to the hypoxic fraction of human brain tumours? *Acta Neurochir* **60** (suppl): 378–380

Franko AJ, Koch CJ, Garrecht BM, Sharplin J and Hughes D (1987) Oxygen concentration dependence of binding of misonidazole to rodent and human tumors *in vitro*. *Cancer Res* **47**: 5367–5376

Franko AJ, Koch CJ and Boisvert DP (1992) Distribution of misonidazole adducts in 9L gliosarcoma tumors and spheroids: implications for oxygen distribution. *Cancer Res* **52**: 3831–3837

Freyer JP and Sutherland RM (1985) A reduction in the *in situ* rates of oxygen growth and glucose consumption of cells in EMT6/Ro spheroids during growth. *J Cell Physiol* **124**: 516–524

Freyer JP, Tustanoff E, Franko AJ and Sutherland RM (1984) In situ oxygen consumption rates of V-79 multicellular spheroids during growth. *J Cell Physiol* **118**: 53–61

Green S, Byar D, Strike T, Alexander JR E, Brooks W, Burger P, Hunt W, Mealey J, Odom G, Paoletti P, Pistenmaa D, Ransohoff J, Robertson J, Selker R, Shapiro W and Smith K (1984) Randomized comparison of BCNU, streptozotocin, radiosensitizer and fractionation of radiotherapy in the postoperative treatment of malignant glioma (Study 7702). *Proc Amer Soc Clin Oncol* **3**: 260

Groshar D, McEwan AJB, Parliament M, Urtasun RC, Golberg LE, Hoskinson M, Mercer JR, Mannan RH, Wiebe LI and Chapman JD (1993) Imaging tumour hypoxia and tumor perfusion. *J Nucl Med* **34**: 885–888

Hochachka PW and Guppy M (1987) *Metabolic Arrest and the Biological Control of Time* Harvard University Press: Cambridge

Horten B, Basler G and Shapiro W (1981) Xenograft of human malignant glial tumors into brains of nude mice. *J Neuropathol Exp Neurol* **40**: 493–511

Ito M, Lammertsma A, Wise R, Bernardi S, Frackowiak R, Heather J, Mckenzie C, Thomas DGT and Jones T (1982) Measurement of regional cerebral blood flow and oxygen utilization in patients with cerebral tumours using $^{15}$O and positron emission tomography: analytical techniques and preliminary results. *Neuroradiology* **23**: 63–74

Jones T, Bigner S, Schold SC, Eng L and Bigner D (1981) Anaplastic human gliomas grown in athymic mice: morphology and glial fibrillary acidic protein expression. *Am J Pathol* **105**: 316–327

Joseph P, Jaiswal AR, Stobbe CC and Chapman JD (1994) The role of specific reductases in the intracellular activation and binding of 2-nitroimidazoles. *Int J Radiat Oncol Biol Phys* **29**: 351–355

Kinter DJH, Fitzpatrick JR JA, Louie JA and Gilboe DD (1984) Cerebral $O_2$ and energy metabolism during and after 30 minutes of moderate hypoxia. *Am J Physiol* **247**: E475–E482

Koch CJ (1984) A 'thin-film' culturing technique allowing rapid gas–liquid equilibrium (6 seconds) with no toxicity to mammalian cells. *Radiat Res* **97**: 434–438

Koch CJ, Stobbe CC and Baer K (1984) Metabolism induced binding of 14C-misonidazole to hypoxic cells: kinetic dependence on oxygen concentration and misonidazole concentration. *Int J Radiat Oncol Biol Phys* **10**: 1327–1331

Kornblith PL, Cummins CJ, Smith BH, Brooks R, Patronas N and Di Chiro G (1984) Correlation of experimental and clinical studies of metabolism by PET scanning. *Prog Exp Tumour Res* **27**: 170–178

Krauseneck P and Muller B (1995) Chemotherapy of malignant brain tumours. In *Malignant Brain Tumours*, Thomas DGT and Graham DI (eds), pp. 329–353. Springer-Verlag: New York

Lammertsma A, Wise R, Cox T, Thomas DGT and Jones T (1985) Measurement of blood flow, oxygen utilization, oxygen extraction ratio and fractional blood volume in human brain tumours and surrounding oedematous tissue. *Br J Radiol* **58**: 725–734

Moulder JE and Rockwell S (1984) Hypoxic fractions of solid tumors: experimental techniques, methods of analysis and a survey of existing data. *Int J Radiat Oncol Biol Phys* **10**: 695–712

Nelson D, Schoenfeld D, Weinstein A, Nelson J, Wasserman T, Goodman R and Carabell S (1983) A randomized comparison of misonidazole sensitized radiotherapy plus BCNU and radiotherapy plus BCNU for treatment of malignant glioma after surgery: preliminary results of an RTOG study. *Int J Radiat Oncol Biol Phys* **9**: 1143–1151

© Cancer Research Campaign 1997

Nelson J, Tsukada Y, Schoenfeld D, Fulling K, Lamarche J and Peress N (1983) Necrosis as a prognostic criterion in malignant supratentorial, astrocytic gliomas. *Cancer* **52**: 550–554

Olive PL, Vikse C and Trotter MJ (1992) Measurement of oxygen diffusion distance in tumor cubes using a fluorescent hypoxia probe. *Int J Radiat Oncol Biol Phys* **22**: 397–402

Parliament MB, Wiebe LI and Franko AJ (1992) Nitroimidazole adducts as markers for tissue hypoxia: mechanistic studies in aerobic normal tissues and tumor cells. *Br J Cancer* **66**: 1103–1108

Rampling R, Cruickshank G, Lewis A, Fitzsimmons S and Workman P (1994) Direct measurement of $pO_2$ distribution and bioreductive enzymes in human malignant brain tumors. *Int J Radiat Oncol Biol Phys* **3**: 427–431

Rana M, Pinkerton H, Thornton H and Nagy D (1977) Heterotransplantation of human glioblastoma multiforme and meningioma to nude mice. *Proc Soc Exp Biol Med* **155**: 85–88

Rockwell S and Moulder JE (1990) Hypoxic fractions of human tumors xenografted into mice: a review. *Int J Radiat Oncol Biol Phys* **19**: 197–202

Shapiro W and Basler G (1979) Chemotherapy of human brain tumors transplanted into nude mice. In *Multidisciplinary Aspects of Brain Tumour Therapy* Paoletti P, Walker M, Butti G and Knerich R (eds), pp. 309–316. Elsevier: Amsterdam

Shapiro W, Basler G, Chernik N and Posner J (1979) Human brain tumor transplantation into nude mice. *J Natl Cancer Inst* **62**, 447–453.

Urtasun R, Band P, Chapman JD, Feldstein M, Mielke B and Fryer C (1976) Radiation and high dose metronidazole in supratentorial glioblastoma. *N Engl J Med* **294**: 1364–1367

Urtasun RC, Koch CJ, Franko AJ, Raleigh JA and Chapman JD (1986) A novel technique for measuring human tissue $pO_2$ at the cellular level. *Br J Cancer* **54**: 453–457

Urtasun RC, Parliament MB, Mcewan AJ, Mercer JR, Mannan RH, Wiebe LI, Morin C and Chapman JD (1996) Measurement of hypoxia in human tumors by non-invasive SPECT imaging of iodoazomycin arabinoside. *Br J Cancer* **74:** 5209–5212

Valk PE, Mathis CA, Prados MD, Gilbert JC and Budinger TF (1992) Hypoxia in human gliomas: demonstration by PET with Fluorine-18-fluoromisonidazole. *J Nucl Med* **33**: 2133–2137

Warburg O (1956) On the origin of cancer cells. *Science* **123**: 309–314

Wilden J, Moore I and Garfield J (1987) Histological factors in the prognosis of malignant gliomas. In *Brain Oncology: Biology, Diagnosis and Therapy*, Chatel M, Darcel F and Pecker J (ed), pp. 243–247. Martinus Nijhoff: Dordrecht

Wise RJS, Thomas DGT, Lammertsma A and Rhodes CG (1984) PET scanning of human brain tumors. *Prog Exp Tumour Res* **27**: 154–169

© Cancer Research Campaign 1997